# Analysis across multiple tumor types provides no evidence that mutant p53 exerts dominant negative activity

**DOI:** 10.1038/s41698-018-0074-x

**Published:** 2019-01-07

**Authors:** Ashkan Shahbandi, James G. Jackson

**Affiliations:** Tulane School of Medicine, Department of Biochemistry and Molecular Biology, 1430 Tulane Avenue, New Orleans, LA 70112 USA

## Abstract

Missense mutations in the *TP53*-binding domain predominate, and >30% of these occur in just eight codons. Dominant negative properties of mutant p53, taken together with the mutation susceptibility of the nucleotides in the codon, are believed to explain the prevalence of specific mutations, including hot spots. We analyzed multiple tumor types and found no difference in clinical characteristics or survival between patients with dominant negative p53 mutant tumors and those with *TP53* mutations that are predicted to be non-dominant negative. The rate tumors underwent loss of heterozygosity in these respective mutation classes was nearly identical, suggesting that presence of stable, mutant protein with predicted dominant negative activity does not reduce selective pressure to inactivate the wild-type allele. Our data suggest all inactivating mutations of *TP53* are equal, and the frequency of dominant negative, hot spot mutations is likely driven more by the relative mutability of the DNA at specific codons.

## Introduction

*TP53* is the most frequently mutated gene in cancer. Many studies over the last 30 years have demonstrated that the majority of pathogenic variants (“mutation” is used synonymously herein) occur in the DNA-binding domain, producing a stable protein shown in various model systems to have dominant negative (DN) and gain-of-function (GoF) activities.^1^ DN activity is derived from the DNA-binding domain mutant protein inhibiting the activity of wild-type (WT) protein in the context of the p53 homotetramer.^2–4^

Various studies have suggested reasons for the prevalence of specific mutations in *TP53*.^5^ Recently, Giacomelli et al.^6^ comprehensively examined functional consequences of *TP53* variants as well as the processes that explain the existence of frequently occurring “hot spot” mutations. Their data demonstrate that a missense mutation in the DNA-binding domain of p53 creates a protein with DN activity and that the probability of acquiring a specific mutation is based both on the functional consequence of the mutation (inactivation, DN activity) and the nucleotide context of the codon.^6^ We examined *TP53* mutations in large human tumor datasets^7^ for evidence of agreement.

## Results

We surmised that if DNA-binding domain DN mutant proteins inactivate the functional protein expressed from the WT allele, then loss heterozygosity (LOH) of this WT allele would not be necessary as often when compared to tumors with null *TP53* mutations that have no effect on WT protein. In other words, there would be less selective pressure to lose the WT *TP53* allele when it is inactivated by the protein made by the mutant allele.

We examined tumor data from three organ sites, ovary, breast, and lung, because each dataset has >300 patients with p53 mutant tumors, clinical information, and LOH data available in cBioportal.^7^ Further, in each of these tumor types, *TP53* mutations are driver events with high variant allele fraction.^8^ We divided tumors into three groups: those with p53 inactivating mutations that have DN activity; those with DN activity and confirmed stability (DN-stable)^9^ and those with mutations that are predicted to create an inactivated protein that is also unable to tetramerize with and inhibit WT p53, and thus not dominant negative (non-DN, includes tetramerization domain, truncating/nonsense, and out of frame indel mutations). After analysis of 306 ovarian, 663 breast, and 672 lung cancer patients, we found *no difference* in frequency of LOH in tumors that had a DN, DN-stable, or a non-DN mutation (Table 1).

**Table 1:** Proportion of loss of heterozygosity (LOH) at *TP53* locus in ovarian, breast, and lung cancer patients with *TP53* mutant tumors

Ovary	LOH	No LOH	Proportion
Mutant	185	121	0.6
DN	111	71	0.61
Non-DN	41	31	0.57
Stable-DN	29	22	0.57
Breast	LOH	No LOH	Proportion
Mutant	553	110	0.83
DN	305	61	0.83
Non-DN	190	38	0.83
Stable-DN	89	23	0.79
Lung	LOH	No LOH	Proportion
Mutant	202	470	0.3
DN	123	285	0.3
Non-DN	57	141	0.29
Stable-DN	10	47	0.18

**Fig. 1:**
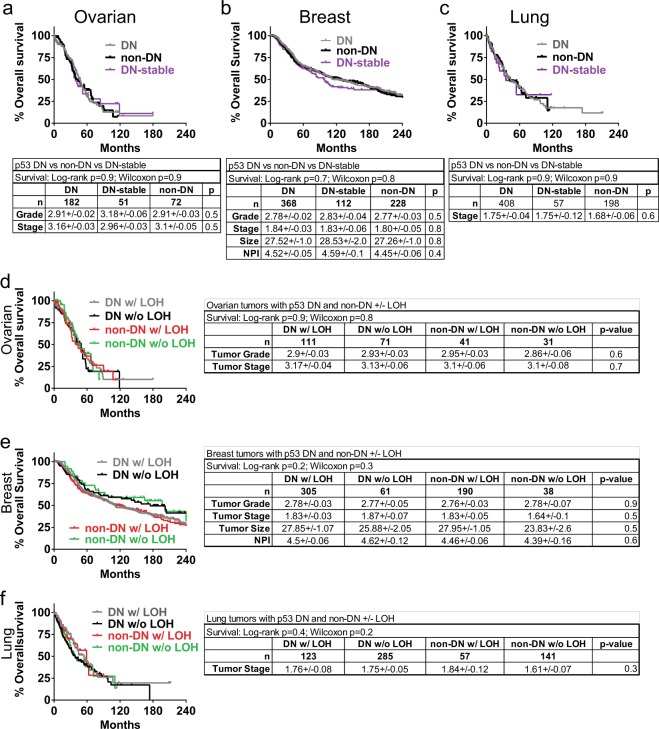
Patient survival and tumor characteristics are identical whether tumors have stable, dominant-negative p53 mutations or non-dominant-negative mutations. Overall survival curves and clinical characteristics of patients with *TP53* mutant ovarian, breast and lung cancers were stratified by mutation type and/or occurrence of loss of heterozygosity (LOH) at *TP53* locus. **a–c** Overall survival and clinical data for ovarian, breast, and lung cancer patients in the TCGA (lung/ovarian) and METABRIC (breast) datasets with DN, non-DN, and DN-stable *TP53* mutant tumors accessed through cBioportal. “DN-stable” refers to DN *TP53* mutations that others have shown to result in a stabilized protein: R175H, G245D/S, R248Q/W/L, R249S, R273C/H/L, and R282W. **d–f** Clinical data for ovarian, breast, and lung cancer patients in the TCGA and METABRIC datasets with DN and non-DN *TP53* mutant tumors stratified by occurrence of LOH at *TP53* locus

Further examination showed no difference in survival whether tumors had DN, DN-stable, or non-DN mutation (Fig. 1a–c), or whether these had undergone LOH (Fig. 1d–f). Characteristics for each tumor type (such as grade, tumor stage, etc) likewise showed no difference depending on type of mutation, or whether the tumor underwent LOH or not (Fig. 1d–f).

Germline variants in *TP53* result in the Li Fraumeni cancer predisposition syndrome. Recent studies showed that patients that harbored a DN mutation in *TP53* had earlier onset of a wide tumor spectrum than those with non-DN.^10^ We examined the IARC database of germline mutation carriers,^11^ and found a similar trend (age of onset for DN was 27.1 years old, *n* = 553; non-DN was 29.0 years old, *n* = 232; *p* = 0.13). When only breast tumors were examined, however, age of onset for DN versus non-DN was identical (35.5 years, *n* = 129; 35.5 years, *n* = 62, respectively, *p* = 1). The proportion of DN versus non-DN variants in Li Fraumeni patients was 0.7, similar to ovary, breast and lung somatic tumors (0.7, 0.6, and 0.7, respectively). These data suggest mutant p53 exerts DN activity in non-breast tumors of germline carriers, however, consistent with Fig. 1b, no evidence of a DN effect was found in breast cancers.

## Discussion

In our analysis of three different tumor types, the data do not support a model whereby the presence of a stable, mutant p53 inactivates the WT protein in DN fashion, or confers on the tumor more aggressive phenotypes that shorten survival in somatic tumorigenesis. However, our findings do support the notion that susceptibility of mutation for certain nucleotides in specific codons is a major determinant of mutation bias.^5,6^ It has previously been posited that the abundance of specific *TP53* mutations occurring in the DNA-binding domain was due to those mutant proteins being selected for during tumorigenesis due to their DN and GoF activities.^5,12^ Mutations that merely inactivate one allele without producing a DN and GoF capable protein would more likely need additional events to inactivate the WT *TP53* allele and promote tumorigenesis, thus explaining why non-DN mutations occur less frequently. Our analysis of 1641 tumors showed that this is unlikely since prevalent DN and the rarer non-DN mutations do not affect tumor phenotype or rate of LOH (Fig. 1 and Table 1). Studies, including Giacomelli et al.,^6^ show data that strongly support this: a decisive factor in the prevalence of DNA-binding domain DN variants was the susceptibility of mutation at that DNA sequence.^13^ Our data from a large sample of diverse tumor types suggest the mutation susceptibility model is correct and perhaps should be given even more weight in explaining the existence of hot spot mutations.

Interestingly, a lack of DN activity by DNA-binding domain mutant proteins is supported by findings in genetically engineered mouse models. Mice with heterozygous knockin DN mutations have identical survival as mice with null alleles.^14,15^ However, DN activity of mutant p53 shortened survival when the level of wild-type p53 protein was only ~10% of normal^16^ or when mutant p53 was highly over-expressed compared to the level of endogenous, wild-type protein.^17^ DN activity may also be exerted in other tumor types not examined here, or during development of non-breast tumors in Li Fraumeni patients.^10^ It is noted, however, that for most or all tumors, even in the context of a DN *TP53* mutation, loss of the WT allele would still result in further reduced p53 activity and likely confer an advantage.

Our findings, taken with the account of mutation frequency offered by others,^6,13^ support a model where all inactivating mutations of *TP53* are essentially “equal”, and the abundance of DN mutations is more likely driven by the DNA context at those codons, rather than selection for those mutant proteins driving tumor phenotypes beyond disabling p53 activity.

## Methods

### Data analysis

METABRIC (breast) and TCGA (lung and ovarian) data were accessed through cBioportal.^7^ Germline *TP53* mutant patient data were accessed through the IARC TP53 Database, and filtered to only include patients diagnosed with Li Fraumeni syndrome. GraphPad Prism software was used to calculate statistical differences in proportion of LOH (Chi-square analysis) and Kaplan–Meier survival curves (Wilcoxon and log-rank (Mantel-cox) tests). Shown below/next to each survival curve is a table containing the sample size in each arm, the mean +/− standard error of the mean (SEM) and *p*-value (calculated via ANOVA and post hoc Tukey’s HSD test) for available tumor characteristics.

### Determination of LOH status at TP53 locus

For tumors from METABRIC dataset, LOH was determined through allele-specific copy-number analysis of tumors (ASCAT). For tumors from TCGA, LOH was determined through analysis of copy-number variation data; tumors with log2 ratio ≤ −0.3 at *TP53* locus were designated as LOH.

## Data Availability

All data were accessed through cBioportal, http://www.cbioportal.org/ or the IARC TP53 Database, http://p53.iarc.fr/TP53GermlineMutations.aspx
